# Adverse events following the first dose of Covishield (ChAdOx1 nCoV-19) vaccination among health workers in selected districts of central and western Nepal: A cross-sectional study

**DOI:** 10.1371/journal.pone.0260638

**Published:** 2021-12-21

**Authors:** Prativa Subedi, Gopal Kumar Yadav, Binod Paudel, Anu Regmi, Prajjwal Pyakurel

**Affiliations:** 1 Department of Internal Medicine, Rolpa District Hospital, Rolpa, Nepal; 2 Department of Internal Medicine, Kalaiya District Hospital, Bara, Nepal; 3 Department of Emergency Medicine, Grahun Primary Hospital, Syangja, Nepal; 4 Department of Medical and Surgical Nursing, Rolpa District Hospital, Rolpa, Nepal; 5 School of Public Health and Community Medicine, B. P. Koirala Institute of Health Sciences, Sunsari, Nepal; Mahidol Oxford Clinical Research Unitl (MORU), THAILAND

## Abstract

**Introduction:**

The study aimed at exploring the adverse events following immunization (AEFI) and their incidences among health workers in three different districts of central and western Nepal following the first dose of Covishield vaccine,. It also aimed at studying the association of AEFI with demographic and clinical characteristics of vaccinees, pre-vaccination anxiety level and prior history of COVID-19 infection (RT- PCR confirmed) status.

**Materials and methods:**

This was a cross-sectional study carried out via face-to-face or telephonic interview among 1006 health workers one week after receiving their first dose of the Covishield vaccine. Incidence of adverse events was calculated in percentage while Chi-square Test was used to check the association of AEFI with independent variables. Logistic regression was used to find out the adjusted odd’s ratio at 95% CI.

**Results:**

Incidence of AEFI was 79.8% with local and systemic AEFI being 68.0% and 59.7% respectively. Injection site tenderness was the commonest manifestation. Local and systemic symptoms resolved in less than one week among 96.8% and 98.7% vaccinees respectively. Females were more likely to develop AEFI than males (AOR = 1.7, 95% CI = 1.2–2.4). Vaccinees aged 45–59 years were 50% less likely to develop AEFI as compared to those aged less than 30 years (AOR 0.5, 95% CI = 0.3–0.8). Most of the vaccinees had not undergone RT-PCR testing for COVID-19 (59.8%). Those who were not tested for COVID-19 prior were 1.5 odds more likely to develop AEFI compared to those who were negative (AOR = 1.5, 95% CI = 1.1–2.1).

**Conclusion:**

More than two-third of the vaccinees developed one or more forms of adverse events, but most events were self-limiting. Females and young adults were more prone to develop AEFI.

## Introduction

COVID-19 is an acute respiratory illness caused by a highly transmissible and pathogenic novel virus named Severe Acute Respiratory Syndrome coronavirus 2 (SARS-CoV-2) [1]. It started as an outbreak in Wuhan, China and was declared a pandemic by World Health Organization (WHO) on March 11, 2020 [2]. As of 11^th^ October 2021, 237,383,711 confirmed cases of COVID-19, and 4,842,716 deaths have been reported to WHO [3]. Meanwhile, the number of confirmed cases in Nepal was 802,861 with 11,243 total deaths [4]. The number of confirmed cases in Nepal was 272,840 with 2,055 deaths as of 16 February 2021, 07:00:00 hours, which amounts to a 194.2% rise in the number of cases and a 447.1% rise in mortality in about eight months [4]. This rise in the number of cases could be mainly due to the porous border with India and the increased transmissibility of new variant of coronavirus which has led to an alarming surge of cases in the second wave [5]. Additionally, poor hand hygiene measures, inappropriate use of masks, inadequate rigor in maintaining social distancing and inability to maintain isolation protocols could have led to the increase of cases in Nepal [6,7].

Various treatment options like remdesivir, antivirals, antimalarials, steroids, cytokine inhibitors, monoclonal antibodies and convalescent plasma therapy have been proposed for the remedy of COVID-19. However, none of them have been found to be effective in curing the disease, albeit clinical benefits have been observed [8]. Herd immunity via mass vaccination is the most promising method to combat the pandemic of COVID-19 although vaccination has not been able to reach all the population [9,10]. A lot of scientific rigor has therefore been put into the invention of the vaccine against coronavirus. COVID-19 vaccine is the fastest developed vaccine in history with the Pfizer-BioNTech vaccine being approved for emergency use on December 2 in UK just a year after the first case was reported in China [11–13]. As of September 16,37 vaccines have completed Phase III trial and 22 vaccines have been approved for use by at least one country [14]. Nearly 5.71 billion COVID-19 vaccines have been administered worldwide until September 13 [15]. However, the efficacy rate of different vaccines against different variants of COVID-19 has been varied [16]. COVID-19 vaccination rolled out in Nepal on 27^th^ January 2020 with frontline workers including health workers and security personnel being vaccinated in the first phase [17]. The vaccine administered was ChAdOx1 nCoV-19 (Covishield vaccine) which is an adenovirus vector vaccine [18]. Inactivated Vero cell vaccine manufactured in China was introduced in the second phase [19]. Covishield vaccine is one of the recombinant vaccines developed by Oxford University and manufactured by Serum Institute of India. AstraZeneca vaccine belongs to the same generic group, although the manufacturers vary; they are considered completely equivalent by WHO Interim guidelines [20].

Some degree of adverse events following immunization (AEFI) has been reported with all of the vaccines developed to date with COVID-19 vaccination being no exception. Following the first dose of the Pfizer BioNTech vaccination, injection site reactions were reported in 65.4% and systemic reactions were reported in 48%, whereas 73.9% injection site reactions and 51.7% systemic reactions were reported after the first dose of Moderna Vaccination [21]. A study conducted using COVID Symptom Study app in the UK showed the incidence of local and systemic reactions as 58.7% and 33.7% respectively following the first dose of ChAdOx1 nCoV-19 vaccine [22]. Another study done among health workers in Korea using the Mobile Vaccine Adverse Events Reporting System (MVAERS) showed an AEFI of 66.1% following the first dose of ChAdOx1 nCoV-19 vaccine [23]. A study done by Shrestha et al in one of the largest tertiary hospitals in central Nepal showed an 85.04% incidence of AEFI following first dose of Covishield vaccination [24]. There is a lack of sufficient evidence regarding the safety profile of various vaccines. Insufficient evidences lead to vaccine hesitancy in public, There are speculations that adequate vaccine coverage might not be achieved despite vaccine availability due to vaccine hesitancy [25].

This study was carried out among a large pool of healthcare workers vaccinated during the first phase in three different districts in central and western Nepal. It aimed to explore the incidence of adverse events following immunization (AEFIs) with the Covishield vaccine, the association of AEFI with vaccinees’ demographic attributes, prior COVID-19 vaccine anxiety and prior COVID-19 infection status.

## Materials and methods

### Design

This was a cross-sectional study carried among frontline health care workers in three different districts (Bara, Rolpa and Syangja) of central and western Nepal.

### Setting

#### General setting

Nepal is a small landlocked country situated in the South East Asia between India and China. Nepal has an area of 147,516 km^2^ and an estimated population of 29,136,808 which constitutes 0.37% of world’s population.

The day, before the vaccination was rolled out in Nepal (26 January 2021, 07:00:00 hours), 269,788 confirmed cases and 2,011 deaths were reported to WHO and the total number of RT-PCR tests done were 2,048,113 [26].

#### Specific setting

This study was carried out in three different districts (Rolpa, Bara and Syangja) of central and western Nepal. Bara is one of the districts in Terai belt and situated in Province no. 2 of central Nepal. Syangja and Rolpa are two hilly districts and are situated in province no. 4 and 5 of western and mid-western Nepal, respectively.

The first phase of vaccination was carried out in district hospitals of these districts under the supervision of respective District Health Offices (DHOs). A medical officer designated as AEFI surveillance officer was responsible to monitor and manage AEFIs. Vaccines were stored in DHOs, and a cold chain was maintained at 2–8^▪^C.

### Study population

All health workers, working in public and private health facilities including doctors, nurses, paramedics, Female Community Health Volunteers (FCHVs) and sanitation workers/office assistants, who had received the first dose of Covishield vaccine, were included. Health workers who were out of the station and those who refused to provide consent were excluded from the study.

### Sample size and sampling technique

Since no studies were conducted during the first dose of vaccination, sample size was calculated considering a prevalence of 50%, 20% allowable error and 95% Confidence Interval by using one proportion formula n=Z2pqL2. The total sample size calculated was 100. The detailed elaboration of sample size calculation is as follows:

n = total sample size
Z = reliability coefficient. Its value for 95% confidence interval is 1.96. So, on squaring it comes out to be 3.84. Hence, we have considered 4 in round off.
p = the proportion in population possessing the characteristics of interest. However, in this study we couldn’t find the prevalence of AEF (p) for sample size calculation, so we kept 50% in the formula to yield maximum value of n.
q = compliment of p = 100−*P* = 100−50 = 50%
L = Permissible error where we have considered 20% of p (20% of 50 = 10) to keep the power of our study at and above 80% at 95% CI.

Hence, $n=\frac{4\times 50\times 50}{10^{2}}=\frac{4\times 2500}{100}=\frac{10000}{100}=100$

**Fig 1:**
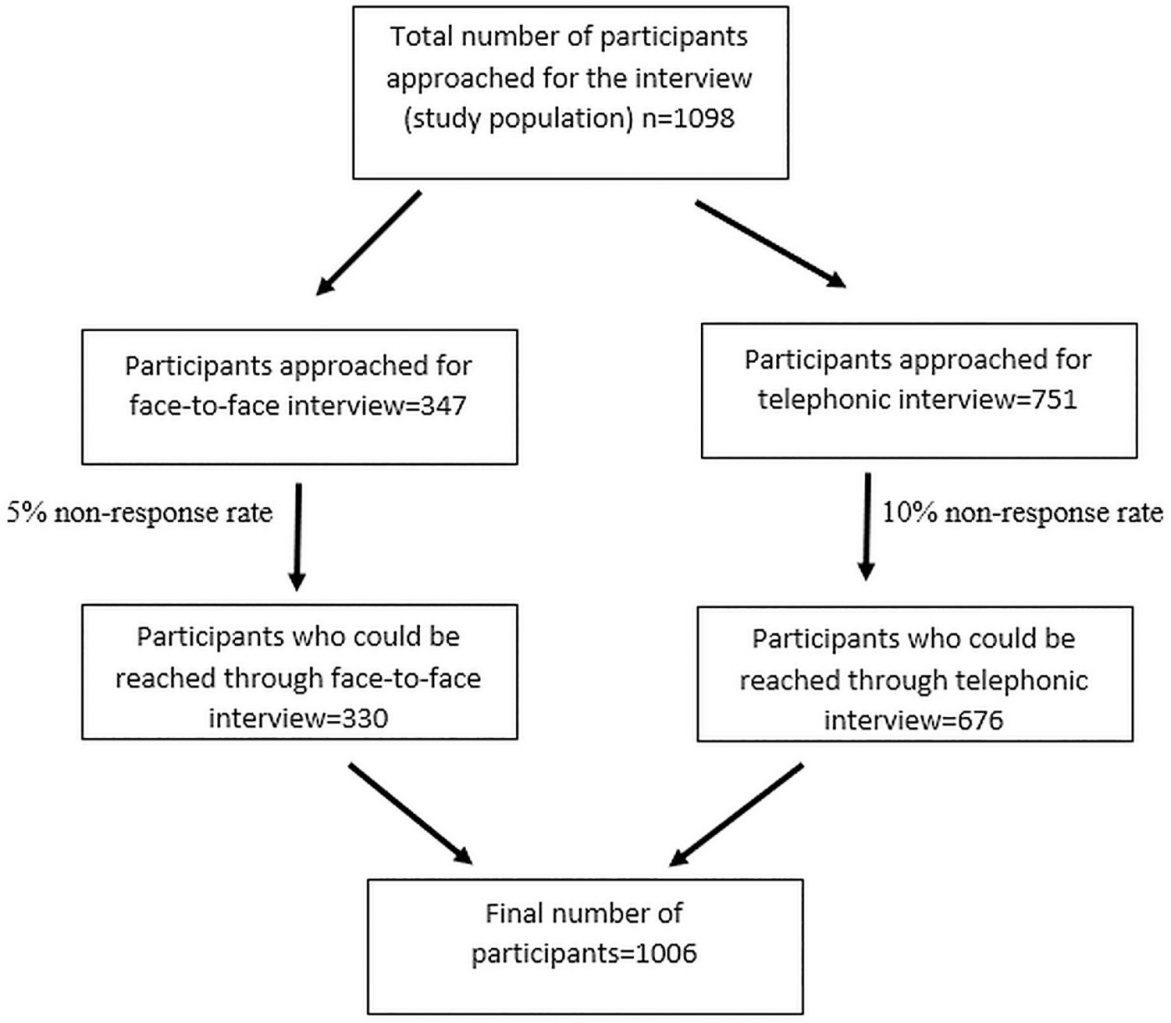
Flow chart showing participants selected in the study.

However, we enrolled 1006 health workers in the final analysis **(Fig 1).** The samples were selected purposively through record review maintained at DHO.

### Variables studied

We considered age, gender, comorbid conditions, pre-vaccination anxiety status, and prior COVID infection status as risk factors; the Covishield vaccine as the exposure; AEFI, medication and hospitalization rates due to AEFI as outcome variables. Content validity was ensured by consulting with infectious disease experts.

### Data collection

Data was collected through semi-structured questionnaires via face-to-face interview and/or by telephonic survey. Basic demographic profile such as age, gender, profession, and telephone number of the health workers were extracted from the DHO of three districts maintained at password protected computer. Face to face interview was conducted by visiting different health facilities with adequate measures for COVID-19 prevention. Whenever there was difficulty in conducting face to face interviews, telephonic interviews were conducted. Data collection was done by the principal investigator and the co-investigators after discussing in the online platform (Zoom) and reviewing the responses every day.

### Data entry and analysis

Data were collected through mobile based application named KoBoCollect v1.30.1 and extracted into Excel sheet. Appropriate commands were used for data cleaning. Entered data was analyzed using Statistical Packages for Social Sciences (SPSS) version 17. Demographic and clinical characteristics were presented in the form of frequency and percentages. AEFI, timing of onset and duration were presented as frequency, percentage, and bar diagram. Bivariate analysis Chi-Square test was used to see the association of various independent variables with the AEFI. A multivariate logistic regression model was fitted to see the association of the AEFI with various independent variables. A p-value cut-off of 0.2 was used to include variables in the regression model. All the data were presented as Odd’s ratio (OR) and Adjusted Odd’s ratio (AOR) at 95% confidence Interval (CI).

### Ethical approval

Ethical approval was taken from the Nepal Health Research council which is the apex body of research in Nepal (Reference no. 2350). Written consent was taken from vaccinees with whom a face-to-face interview was done. Due to difficult topography and Covid-19 imposed mobility restrictions, a telephonic interview was conducted instead of face-to-face interview for some participants. All the participants were explained about the objectives of this research and the possible risks and benefits of participating in the study before the interview. They were also assured that their participation was entirely voluntary and they could quit the interview at any stage. Written informed consent was obtained for a face-to-face interview. For a telephonic interview, verbal consent was taken and recorded during the beginning of telephonic interview.

#### Operational definition

Adverse events following immunization (AEFI): It refers to any medical response (local or system) that occurs following immunization that may not necessarily have a causal relationship with the administration of vaccine.

Anxiety status: Measurement of level of anxiety using a 4 point Likert scale for Anxiety (known as GAD-2 scale): 1 = not at all, 2 = Several days, 3 = more than half the days and 4 = nearly every day.

Allergic history: The history of reactions like itching, rashes or shortness of breath following intake of any food or drugs in the past.

Myalgia/Body ache: Sensation of pain all over the body

Fatigue: Feeling of weakness and tiredness

## Results

Out of 1098 participants, 1006 consented to participate in the study. The response rates for face-to-face and telephonic interviews were 95% and 90% respectively. There was the predominance of female participants (58%). The mean age± SD of the participants was 34.5±10.8 years with most participants belonging to age group of < 30 years (41.8%). Most of the participants were paramedics (35.5%) followed by FCHVs (25.4%) **(Table 1).**

**Table 1:** Demographic characteristics of the participants receiving 1^st^ dose of Covishield vaccine (n = 1006).

Characteristics	Participants	Percentage (%)
**Gender**		
Male	423	42.0
Female	583	58.0
**Age groups (Years)**		
<30	420	41.7
30–44	372	37.0
45–59	193	19.2
≥60	21	2.1
**Mean age± SD = 34.5±10.8 years**		
**Professions**		
Doctors	70	7.0
Nurses	202	20.1
Paramedics	357	35.5
FCHVs	256	25.4
Office Assistants	121	12.0

About 9.5% of participants have one or more co-morbidities. Hypertension (59.4%) and Diabetes (30.2%) were commonly identified comorbidities. Prior to the 1^st^ dose of vaccination 5.3% of total participants tested positive for COVID-19 detected through RT-PCR while 34.8% were tested negative. The remaining fraction had not undergone RT-PCR testing. Regarding the pre-vaccination anxiety level, 10% of the participants have moderate to very much anxiety level before vaccination. More than one-third of the participants (35.7%) took medicine and paracetamol was the most commonly used medication which constituted 71.6% of total medication. 8.6% of vaccinees had to take leave due to AEFI while 1.9% required hospital visit/hospitalization. When inquired regarding the perception of the safety of vaccine, 69.9% of the vaccinees considered the Covishield vaccine to be safe. 29.6% mentioned that they were not sure of its safety and 0.5% mentioned that they were unsafe **(Table 2).**

**Table 2:** Clinical characteristics of the participants receiving 1^st^ dose of Covishield vaccine (n = 1006).

Characteristics	Participants	Percentage (%)
**Co-morbidities Status**		
No	910	90.5
Yes	96	9.5
**Types of Co-morbidities** ^**1**^		
Hypertension	57	59.4
Diabetes	29	30.2
COPD	5	5.2
Thyroid disorders	16	16.7
Heart Disease	4	4.2
Dyslipidemia	4	4.2
Psychiatric Illness	4	4.2
Rheumatoid arthritis	2	2.0
HIV	1	1.0
**COVID-19 RT-PCR Status** ^**2**^		
Not tested	603	59.9
Negative Status	350	34.8
Positive Status	53	5.3
**Anxiety status** ^**3**^		
Not at all	432	42.9
Several days	462	45.9
More than half the days	95	9.4
Nearly every day	17	1.7
**Allergy history** ^**4**^		
No	926	92.0
Yes	19	1.9
Can’t remember	61	6.1
**Medication for reactions** ^**5**^		
No	647	64.3
Yes	359	35.7
**Leave following reactions** ^**5**^		
No	920	91.4
Yes	86	8.6
**Hospital visit/Hospitalization** ^**5**^		
No	987	98.1
**Yes**	**19**	**1.9**
**Perception of vaccine safety**		
No	6	0.6
Yes	702	69.8
Not sure	**298**	**29.6**

^1^Multiple response answer.

^2^Prior to receiving the first dose of Covishield vaccination.

^3^Pre-vaccination anxiety level.

^4^Pre-vaccination reactions to drugs or other vaccines.

^5^Post-vaccination events.

The incidence of AEFI among participants receiving 1^st^ dose of Covishield vaccination was 79.4%. Majority of participants have both local and systemic reactions (48.3%). Injection site tenderness (60.3%) and fever (38.2%) were the most common local and systemic reactions. Most of the participants (16.3%) had a combination of two reactions which may be either local and/or systemic **(Table 3).**

**Table 3:** Incidence of AEFI among participants following 1^st^ dose of Covishield vaccine (n = 1006).

Characteristics	Participants	Percentages (%)
**Adverse Events**		
Presence	799	79.4
Absence	207	20.6
Total	1006	100
**Types of adverse events**		
Local	198	19.7
Systemic	115	11.4
Both local and systemic	486	48.3
**Local reactions** ^**1**^		
Injection site tenderness	607	60.3
Injection site pain	427	42.4
Injection site swelling	23	2.3
Injection site redness	6	0.60
**Systemic reactions** ^**2**^		
Fever	384	38.2
Body ache/Myalgia	*324*	32.2
Headache	290	28.9
Chills	222	22.1
Fatigue	176	17.5
Dizziness	121	12.0
Drowsiness	110	11.0
Nausea and/or vomiting	45	4.5
Irritable Mood	33	3.2
Anxiety	11	0.9
Fainting attack	5	0.5
Diarrhoea	7	0.7
Rashes	2	0.2
Shortness of breath	1	0.1
**No. of Reactions** ^**3**^		
0	207	20.6
1	153	15.2
2	164	16.3
3	117	11.6
4	130	12.9
5	93	9.2
>5	142	14.1

^1^Participants had one or a combination of local reactions (Multiple responses).

^2^Participants had one or a combination of systemic reactions (Multiple responses).

^3^Include either local or systemic reactions.

**Fig 2:**
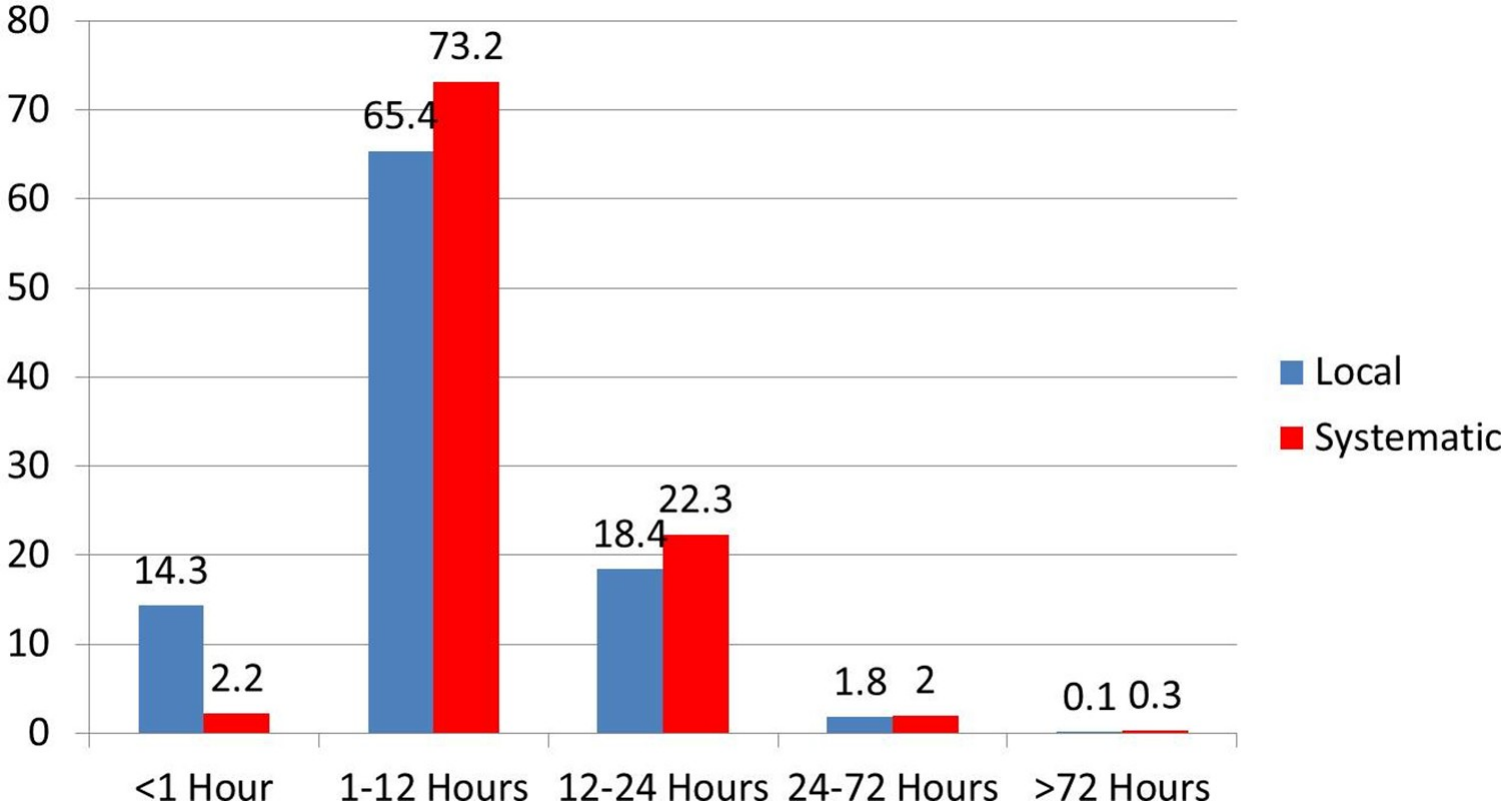
Timing of onset of local and systemic reaction following 1^st^ dose of Covishield vaccine.

Majority of the participants developed local (65.4%) and systemic (73.2%) reactions within 1–12 hours following vaccination. However, the reaction subsided in less than 72 hours **(Fig 2).**

**Fig 3:**
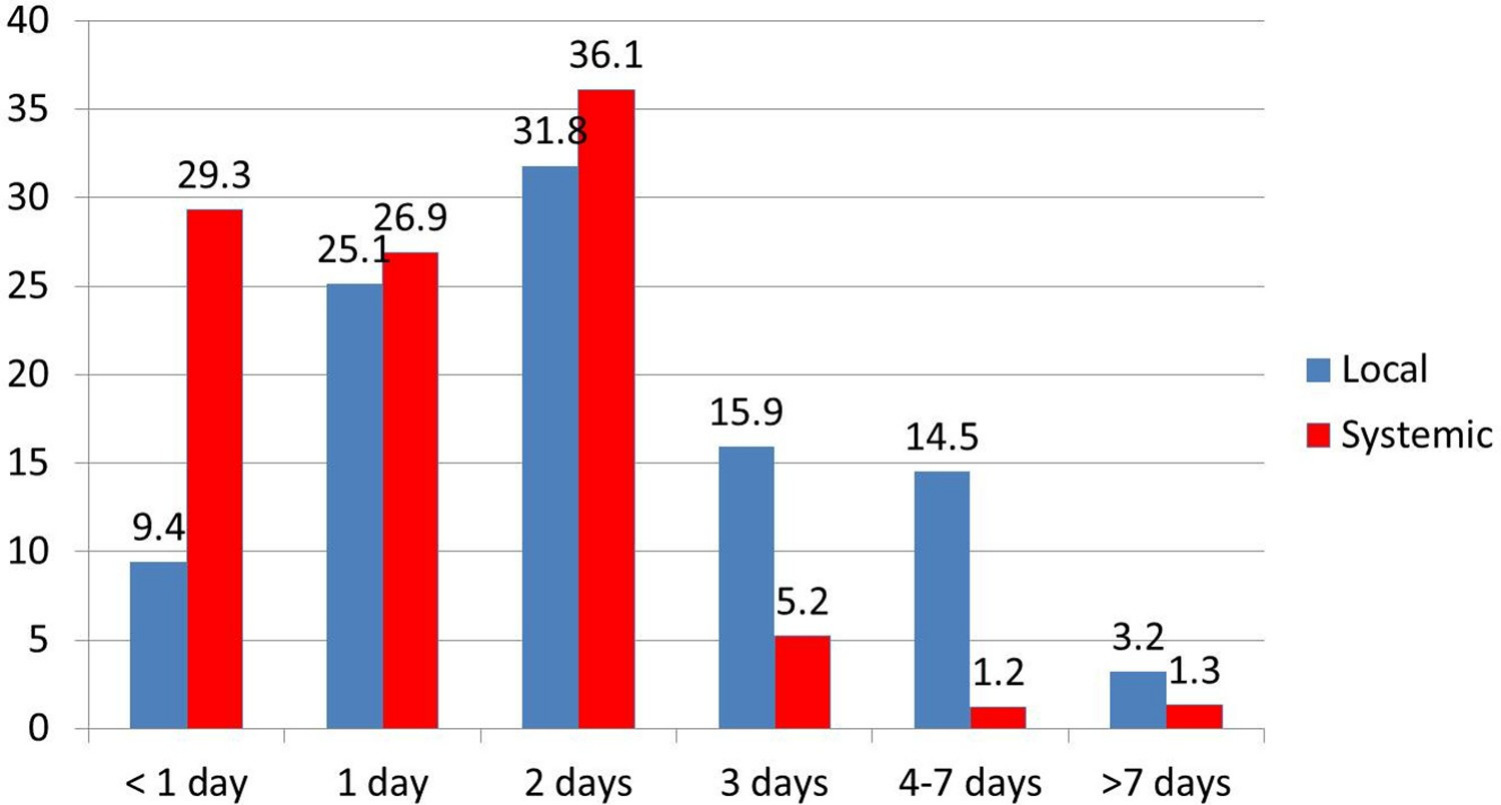
Duration of subsidence of local and systemic reaction following 1^st^ dose of Covishield vaccine.

Local (31.9%) and systematic (36.1%) reactions of the majority of the participants subsided in 2 days which subsequently got negligible after 7 days **(Fig 3).**

Those variables whose P value is < 0.2 were considered for multivariate analysis. In the multivariate analysis, those who were aged 45–59 years were 50% less likely to develop AEFI compared to those who were < 30 years. (AOR = 0.5, 95% CI = 0.3–0.8). Females were 1.9 odds more likely to develop AEFI compared to males (AOR = 1.7, 95% CI = 1.2–2.4). Similarly, those who were not tested for COVID-19 prior were 1.5 odds more likely to develop AEFI compared to those who were negative (AOR = 1.5, 95% CI = 1.1–2.1). Those who have pre- vaccination anxiety were 1.6 odds more likely to develop AEFI (AOR = 1.6, 95%CI = 1.2–2.2) **(Table 4).**

**Table 4:** Association of various demographic and clinical parameters with AEFI (n = 1006).

Parameters	Adverse Events []		OR	95% CI	AOR	95% CI
	No (%)	Yes (%)				
**Age (years)**						
<30	79(38.2)	341(42.7)	1		1	
30–44	67(31.4)	305(38.2)	1.1	0.7–1.5	1.1	0.8–1.6
45–59	55(26.6)	138(17.3)	0.6	0.4–0.9	**0.5**	**0.3–0.8**
≥60	6(2.9)	15(1.9)	0.6	0.2–1.5	0.6	0.2–1.5
**Gender**						
Male	112(26.5)	311(73.5)	1		1	
Female	95(16.3)	488(83.7)	1.9	1.4–2.5	**1.7**	**1.2–2.4**
**Comorbidity**						
**Absent**	184(20.2)	726(79.8)	1			
Present	23(24.0)	73(76.0)	0.8	0.5–1.3		
**COVID RT-PCR**^**1**^ **Status**^**1**^						
Negative	88(25.1)	262(74.9)	1		1	
Positive	15(28.3)	38(71.7)	0.9	0.4–1.6	0.9	0.4–1.7
Not tested	104(17.2)	499(82.8)	1.6	1.2–2.1	**1.5**	**1.1–2.1**
**Preanxiet** **y** ^**2**^						
No	109(25.2)	323(74.8)	1		1	
Yes	98(17.1)	476(82.9)	1.6	1.2–2.2	**1.6**	**1.2–2.2**
**Allergy** ^**3**^						
No	194 (21)	732 (79)	1		1	
Yes	0 (0)	19 (100)	-		-	
No Recall remember	13 (21.3)	48 (78.7)	0.9	0.5–1.8	0.9	0.5–1.9

^1^Pre vaccination COVID-19 RT-PCR Status [Neg. = Negative and Posi. = Positive].

^2^Prevaccination anxiety status.

^3^Pre-vaccination reactions to drugs or other vaccines.

*Adjusted for age, gender, COVID RT-PCR Status, pre-anxiety status and allergy status.

## Discussion

In our study, the incidence of AEFI following the first dose of Covishield vaccination was found to be 79.4%. This finding is quite similar to a study done by Shrestha et al at one of the largest tertiary hospitals of Nepal which reported an 85.0% incidence of AEFI following the first dose of the same vaccine [24]. Another study done among health workers in Korea using the Mobile Vaccine Adverse Events Reporting System (MVAERS) showed a considerably high AEFI of 98.1% following the first dose of ChAdOx1 nCoV-19 vaccine [24]. One possible explanation on this significant incidence could be the fact that this survey was based on a passive reporting system. So, only those developing AEFI tended to report. This is supported by the fact that out of 1,503 health care workers who were vaccinated, the data of 994 vaccinees were only reported in the MVAERS.

The frequency of local reactions was similar to systemic reactions (ratio 1.1), and almost half of vaccinees (48.3%) had both local and systemic reactions. The proportion of vaccinees developing local and systemic reactions was similar in other studies too [22,23]. Amongst those developing AEFI, most of the vaccinees had 2 or more categories of reactions, regardless of whether it is a local or systemic one. Injection site tenderness was the commonest local symptom and fever was the commonest local symptom. In some studies, myalgia is more common than fever [27]. Most cases of fever, myalgia and local site pain have improved with paracetamol alone. Paracetamol was also the most commonly used medication in most of the previous studies [28,29]. According to our study, common symptoms (incidence rate more than 10%) were injection site tenderness, injection site pain, myalgia, fever, headache, chills, dizziness, and drowsiness. Nausea/vomiting, injection site swelling, and irritable mood were common symptoms (incidence between 1 to 10%). Diarrhea, rashes, and shortness of breath were uncommon symptoms (incidence less than 1%). These findings are very similar to the study by Shrestha et al except that dizziness which is a very common AEFI in our study is a common AEFI in their cohort [24]. A study done among health care workers in three university hospitals of Korea showed that systemic reactions following vaccination were more common after ChAdOx1 nCoV-19 vaccine compared to Pfizer but there was no difference in terms of local reaction [27]. Most of the symptoms started within 12 hours and resolved in less than 3 days.

Most of the cases in our study were mild to moderate. None of them required long-term hospitalization and no deaths were reported within a week. ChAdOx1 nCoV-19 vaccine has been linked to thromboembolic events and clotting abnormalities [30,31]. Astrazaneca vaccine was suspended in many countries due to thromboembolic risk [32]. No cases suggestive of deep vein thrombosis, focal neurological deficit, cardiac event and bleeding were reported from our study participants within one week following vaccination. This, being a cross-sectional study, however, the long term sequelae after vaccination could not be studied.

Compared to vaccinees aged less than 30 years, those aged 30–44 years were found to have a greater risk of developing AEFI (AOR 1.1) while those aged 44–59 years and more than 60 years were found to be at a lesser risk (AOR 0.5 and 0.6 respectively). There is no clear consensus on whether age is a determinant of AEFI. A randomized clinical trial in UK showed a lower reactogenicity with lower side effect profile in older adults than young adults [33] while few studies report greater AEFI rates among young adults compared to the older ones [22,23]. Few previous studies exhibit no significant association between age and AEFI [24].

Females were found to be 73% more likely to develop AEFI compared to males. Female preponderance to AEFI has been reported in many similar studies [22,34]. This could be potentially due to the fact that women are considered to have a more robust immune response and mount greater cell-mediated as well as humoral immune response following antigenic stimulation by vaccination or infection when compared to males [35]. A study has shown that antibody titer following vaccination with H1N1 vaccine was greater among females compared to males and the titer was directly linked with the level of serum estradiol [36].

Comorbid conditions did not play in terms of AEFI incidence in our study as in most other similar studies [22,24,37]. A study done among health care workers in Kerala showed an increased risk of AEFI among individuals with Bronchial Asthma but no statistically significant association with other comorbidities like diabetes, hypertension, heart disease or immunological disorder [37].

More than half (57.1%) of the vaccinees had some level of anxiety before getting vaccinated. In fact, anxiety over a newly launched vaccine had led to significant vaccine hesitancy and many health workers did not get vaccinated in the first phase [25]. AEFI was found to be significantly associated with pre-vaccination anxiety status. Anxiety-related stress response can be a likely cause; but since it is a diagnosis of exclusion, no definitive statement can be made unless other causes are ruled out [38]. All vaccinees who had any history of allergy in the past developed one or more adverse events after vaccination. However, none of them developed life-threatening anaphylaxis. A recent research in JAMA network states that even highly allergic individuals can take Pfizer vaccine; however vaccination should be strictly under medical supervision and requires special precautions [39].

The rate of hospital visit is quite low [1.9%] in our study. There could be two-fold explanations for this fact. It could be either because the symptoms were mild and not severe enough to require hospital visit. The other likely reason could be that since this study was done among health workers, they were themselves aware of the possible side effects and were capable of self-medication. The second explanation is supported by the fact that 35.7% mentioned taking medications while only 1.9% reported hospital visits. The rate of self-medication was 55.6% in the study by Shrestha et.al which is quite high as compared to our study [24].

During our assessment of the perception of safety of the vaccine, two-thirds of the vaccinees considered the Covishield vaccine to be safe, while almost one-third mentioned that they were not sure of its safety. The significant proportion of uncertainty could be because this was a new vaccine and health workers were the first to be vaccinated. But despite the uncertainty, most of the vaccinees [98.7%] showed their willingness to get the second dose. This can be because health workers are better aware of the process of immunogenicity of vaccine and that incomplete administration could lead to vaccine failure.

### Strength and limitations

This is the first study in Nepal related to AEFI that covers a wide socio-geographic and demographic setting.

- Data was obtained via investigator-led interview method in contrast to most other studies that are done by application or mobile-based reporting system.
- Data was collected from the respondents one week following vaccination and hence there are chances of recall bias.
- This is a cross sectional study therefore long term sequelae of COVID vaccine could not be studied.
- Large sample size and wide geographic and demographic coverage make our data more representative of the population. However, this research was conducted among a pool of health workers only. The level of knowledge regarding vaccine, possible AEFI and medications are definitely higher among health workers as compared to general public. This could limit the generalizability of our study findings.

## Conclusion

More than two-thirds of vaccinees have reported one or other type of adverse reactions after vaccination, but most of the reactions were nonserious and self-limiting. Females and young adults were more likely to develop AEFI. Comorbidity had no association with AEFI. Since majority of the vaccinees were not tested for Covid infection by RT-PCR technique, the relation of AEFI with prior infection could not be firmly ascertained.

Covishield being the new vaccine, there could be higher vaccine hesitancy due to fear of adverse events. Our study showed that none of the adverse events were serious and most of the AEFI resolved within one week. This could alleviate vaccine hesitancy and increase vaccination coverage among public.

## Supporting information

S1 File COVID-19 questionnaire version 12 PBG. (XLSX)

## Abbreviations

AEFI: Adverse events following immunization
DHO: District Health Office
FCHV: Female Community Health Volunteer
MVAERS: Mobile Vaccine Adverse Events Reporting System
SARS-CoV-2: Severe Acute Respiratory Syndrome Coronavirus 2
RT-PCR: Real Time- Polymerase Chain Reaction
WHO: World Health Organization

## References

[1] Hu B, Guo H, Zhou P, Shi Z-L. Characteristics of SARS-CoV-2 and COVID-19. Nat Rev Microbiol 19. 2021;141–154. Available from: doi: 10.1038/s41579-020-00459-7 33024307 PMC7537588

[2] Cucinotta D, Vanelli M. WHO declares COVID-19 a pandemic. Acta Biomedica. 2020; 91(1):157–160. Available from: https://pubmed.ncbi.nlm.nih.gov/32191675/. doi: 10.23750/abm.v91i1.9397 32191675 PMC7569573

[3] WHO Coronavirus (COVID-19) Dashboard | WHO Coronavirus (COVID-19) Dashboard With Vaccination Data. Available from: https://covid19.who.int/.

[4] Nepal: WHO Coronavirus Disease (COVID-19) Dashboard With Vaccination Data Available from: https://covid19.who.int/region/searo/country/np.

[5] Kansakar S, Dumre SP, Raut A, Huy NT. From lockdown to vaccines: challenges and response in Nepal during the COVID-19 pandemic. Lancet Respir Med. 2021 Jul; 9(7):694–695.Available from: https://www.thelancet.com/journals/lanres/article/PIIS2213-2600(21)00208-3/fulltext. 33932347 10.1016/S2213-2600(21)00208-3 PMC8081397

[6] COVID-19 and hygiene in Nepal | WaterAid Global. Available from: https://www.wateraid.org/global-covid-19-response/nepal.

[7] Rayamajhee B, Pokhrel A, Syangtan G, Khadka S, Lama B, Rawal LB, et al. How Well the Government of Nepal Is Responding to COVID-19? An Experience From a Resource-Limited Country to Confront Unprecedented Pandemic. Front Public Heal. 2021 Feb 17; 9:597808. Available from: doi: 10.3389/fpubh.2021.597808 33681124 PMC7925847

[8] Dong Y, Shamsuddin A, Campbell H, Theodoratou E. Current COVID-19 treatments: Rapid review of the literature. J Glob Health. 2021 Apr 24;11:10003. Available from: doi: 10.7189/jogh.11.10003 33959261 PMC8068411

[9] Frederiksen LSF, Zhang Y, Foged C, Thakur A. The Long Road Toward COVID-19 Herd Immunity: Vaccine Platform Technologies and Mass Immunization Strategies. Frontiers in Immunology. 2020 July 21;11:1817. Available from: doi: 10.3389/fimmu.2020.01817 32793245 PMC7385234

[10] Vignesh R, Shankar EM, Velu V, Thyagarajan SP. Is Herd Immunity Against SARS-CoV-2 a Silver Lining? Frontiers in Immunology. 2020 Sep 30;11:2570. Available from: doi: 10.3389/fimmu.2020.586781 33101320 PMC7554232

[11] Ball P. The lightning-fast quest for COVID vaccines—and what it means for other diseases. Nature. 2021 Jan 1;589(7840):16–8. Available from: doi: 10.1038/d41586-020-03626-1 33340018

[12] WHO issues its first emergency use validation for a COVID-19 vaccine and emphasizes need for equitable global access. Available from: https://www.who.int/news/item/31-12-2020-who-issues-its-first-emergency-use-validation-for-a-covid-19-vaccine-and-emphasizes-need-for-equitable-global-access.

[13] U.K. Approves Pfizer Coronavirus Vaccine, a First in the West—The New York Times. Available from: https://www.nytimes.com/2020/12/02/world/europe/pfizer-coronavirus-vaccine-approved-uk.html.

[14] Vaccines–COVID19 Vaccine Tracker. Available from: https://covid19.trackvaccines.org/vaccines/.

[15] COVID-19 vaccine doses by country. Statista. Available from: https://www.statista.com/statistics/1194934/number-of-covid-vaccine-doses-administered-by-county-worldwide/.

[16] Olliaro P, Torreele E, Vaillant M. COVID-19 vaccine efficacy and effectiveness-the elephant (not) in the room. The Lancet Microbe. 2021 Apr 20;0(0). Available from: doi: 10.1016/S2666-5247(21)00069-0 33899038 PMC8057721

[17] Bhattarai S, Dhungana J. A million-dose success for Nepal: insights from Nepal’s national vaccine deployment plan against COVID-19. J Travel Med. 2021 Mar 1;2021:1–3. Available from: doi: 10.1093/jtm/taab027 33668060 PMC7989250

[18] Loo KY, Letchumanan V, Ser HL, Teoh SL, Law JWF, Tan LTH, et al. COVID-19: Insights into potential vaccines. Microorganisms. 2021 Mar 15:1–19. Available from: doi: 10.3390/microorganisms9030605 33804162 PMC8001762

[19] Jay N Shah. The ‘Vero Cell’ COVID-19 vaccine rollout in Nepal: What we know about the Chinese vaccine development and access? Journal of Patan Academy of Health Sciences. Available from: 10.3126/jpahs.v8i1.36768.

[20] Interim recommendations for use of the ChAdOx1-S [recombinant] vaccine against COVID-19 (AstraZeneca COVID-19 vaccine AZD1222 Vaxzevria™, SII COVISHIELD™). Available from: https://www.who.int/publications/i/item/WHO-2019-nCoV-vaccines-SAGE_recommendation-AZD1222-2021.1.

[21] Chapin-Bardales J, Gee J, Myers T. Reactogenicity following Receipt of mRNA-Based COVID-19 Vaccines. JAMA—Journal of the American Medical Association. 2021 Apr 5. Available from: doi: 10.1001/jama.2021.5374 33818592

[22] Menni C, Klaser K, May A, Polidori L, Capdevila J, Louca P, et al. Vaccine side-effects and SARS-CoV-2 infection after vaccination in users of the COVID Symptom Study app in the UK: a prospective observational study. Lancet Infect Dis. 2021 Apr 27; Available from: doi: 10.1016/S1473-3099(21)00224-3 33930320 PMC8078878

[23] Jeon M, Oh CE, Lee J. Adverse Events Following Immunization Associated with Coronavirus Disease 2019 Vaccination Reported in the Mobile Vaccine Adverse Events Reporting System. 2021 May 03;36(17):e114. Available from: doi: 10.3346/jkms.2021.36.e114 33942578 PMC8093606

[24] Shrestha Shrijana, Ranjan P Devbhandari Ashis Shrestha, Aryal Sushant, Rajbhandari Piyush, Shakya Binnam, et al. Adverse events following the first dose of ChAdOx1 nCoV-19 (COVISHIELD) vaccine from in the first phase of vaccine roll out in Nepal. Journal of Patan Academy of Health Sciences. 2021 Apr;8(1):9–17. Available from: 10.3126/jpahs.v8i1.36242.

[25] Dror AA, Eisenbach N, Taiber S, Morozov NG, Mizrachi M, Zigron A, et al. Vaccine hesitancy: the next challenge in the fight against COVID-19. Eur J Epidemiol. 2020;35(8):775–9. Available from: doi: 10.1007/s10654-020-00671-y 32785815 PMC8851308

[26] Situation Update #41—Coronavirus Disease 2019 (COVID-19) WHO Country Office for Nepal. Available from: https://cdn.who.int/media/docs/default-source/nepal-documents/novel-coronavirus/who-nepal-sitrep/-41_weekly-who-nepal-situation-updates.pdf?sfvrsn=85f688d_5.

[27] Song JY, Cheong HJ, Kim SR, Lee SE, Kim SH, Noh JY, et al. Early Safety Monitoring of COVID-19 Vaccines in Healthcare Workers. J Korean Med Sci. 2021 Apr 19;36(15):e110. Available from: doi: 10.3346/jkms.2021.36.e110 33876589 PMC8055510

[28] Sah R, Shrestha S, Mehta R, Sah SK, Raaban AA, Dharma K, et al. AZD1222 (Covishield) vaccination for COVID-19: Experiences, challenges, and solutions in Nepal. Travel Medicine and Infectious Disease. 2021 Feb 10; 40:101989. Available from: doi: 10.1016/j.tmaid.2021.101989 33578045 PMC7872846

[29] Srivastava RK, Ish P. COVID-Vaccination Group S. The initial experience of COVID-19 vaccination from a tertiary care centre of India. Monaldi Arch Chest Dis. 2021 Mar 31; doi: 10.4081/monaldi.2021.1816 Available from: 10.4081/monaldi.2021.1816. 33794595

[30] Greinacher A, Thiele T, Warkentin TE, Weisser K, Kyrle PA, Eichinger S. Thrombotic Thrombocytopenia after ChAdOx1 nCov-19 Vaccination. N Engl J Med. 2021 Apr 9; 384:2092–2101. Available from: doi: 10.1056/NEJMoa2104840 33835769 PMC8095372

[31] Pottegård A, Lund LC, Karlstad Ø, Dahl J, Andersen M, Hallas J, et al. Arterial events, venous thromboembolism, thrombocytopenia, and bleeding after vaccination with Oxford-AstraZeneca ChAdOx1-S in Denmark and Norway: Population based cohort study. BMJ. 2021 May 5; 373: n1114. Available from: doi: 10.1136/bmj.n1114 33952445 PMC8097496

[32] Wise J. Covid-19: European countries suspend use of Oxford-AstraZeneca vaccine after reports of blood clots. BMJ. 2021 Mar 11; 372:n699. Available from: doi: 10.1136/bmj.n699 33707182

[33] Ramasamy MN, Minassian AM, Ewer KJ, Flaxman AL, Folegatti PM, Owens DR, et al. Safety and immunogenicity of ChAdOx1 nCoV-19 vaccine administered in a prime-boost regimen in young and old adults (COV002): a single-blind, randomised, controlled, phase 2/3 trial. The Lancet. 2020 Nov 18; 396(10267):1979–1993. Available from: 10.1016/S0140-6736(20)32466-1. PMC7674972 33220855

[34] Jayadevan R, Shenoy R, Ts A. Survey of symptoms following COVID-19 vaccination in India. medRxiv. 2021 Feb 12; 2021.02.08.21251366. Available from: 10.1101/2021.02.08.21251366.

[35] Fink A. L., & Klein S. L. Sex and Gender Impact Immune Responses to Vaccines Among the Elderly. Physiology (Bethesda). 2015 Nov; 30(6):408–416. Available from: doi: 10.1152/physiol.00035.2015 26525340 PMC4630198

[36] Potluri T, Fink AL, Sylvia KE, Dhakal S, Vermillion MS, vom Steeg L, et al. Age-associated changes in the impact of sex steroids on influenza vaccine responses in males and females. npj Vaccines. 2019 Dec 1;4(1):1–12. Available from: 10.1038/s41541-019-0124-6. 31312529 PMC6626024

[37] Mathew T, Harikumaran Nair GS, Lathika Rajasekharan Nair G, Rajee Alex M. Adverse events and their association with comorbidities after first and second doses of Covishield vaccination among healthcare workers of Government owned medical colleges in Kerala. medRxiv. 2021 May 25;2021.05.19.21257317. Available from: 10.1101/2021.05.19.21257317.

[38] Palacios R. Considerations on immunization: Anxiety-related reactions in clusters. Colomb Med (Cali). 2014 Jul 1;45(3):136–40. Available from: https://colombiamedica.univalle.edu.co/index.php/comedica/article/view/1711/2463. 25386041 PMC4225792

[39] Shavit R, Maoz-Segal R, Iancovici-Kidon M, Offengenden I, Yahia SH, Maayan DM. *JAMA Netw Open*. 2021;4(8):e2122255. Avilable from: doi: 10.1001/jamanetworkopen.2021.22255 34463744 PMC8408666

